# TRPV1 Activation Antagonizes High-Fat Diet-Induced Obesity at Thermoneutrality and Enhances UCP-1 Transcription via PRDM-16

**DOI:** 10.3390/ph17081098

**Published:** 2024-08-21

**Authors:** Padmamalini Baskaran, Noah Gustafson, Nicolas Chavez

**Affiliations:** 1 College of Pharmacy, Howard University, Washington, DC 20059, USA; 2 School of Pharmacy, University of Wyoming, Wyoming, Laramie, WY 82071, USA; ngustaf2@uwyo.edu (N.G.); nic.chavez.29@gmail.com (N.C.)

**Keywords:** thermoneutrality, TRPV1, UCP-1, PRDM-16

## Abstract

Body weight is a balance between energy intake and energy expenditure. Energy expenditure is mainly governed by physical activity and adaptive thermogenesis. Adaptive dietary thermogenesis in brown and beige adipose tissue occurs through mitochondrial uncoupling protein (UCP-1). Laboratory mice, when housed at an ambient temperature of 22–24 °C, maintain their body temperature by dietary thermogenesis, eating more food compared to thermoneutrality. Humans remain in the thermoneutral zone (TNZ) without expending extra energy to maintain normal body temperature. TRPV1 activation by capsaicin (CAP) inhibited weight gain in mice housed at ambient temperature by activating UCP-1-dependent adaptive thermogenesis. Hence, we evaluated the effect of CAP feeding on WT and UCP-1^−/−^ mice maintained under thermoneutral conditions. Our research presents novel findings that TRPV1 activation by CAP at thermoneutrality counters obesity in WT mice and promotes PRDM-16-dependent UCP-1 transcription. CAP fails to inhibit weight gain in UCP-1^−/−^ mice housed at thermoneutrality and in adipose tissue-specific PRDM-16^−/−^ mice. In vitro, capsaicin treatment increases UCP-1 transcription in PRDM-16 overexpressing cells. Our data indicate for the first time that TRPV1 activation counters obesity at thermoneutrality permissive for UCP-1 and the enhancement of PRDM-16 is not beneficial in the absence of UCP-1.

## 1. Introduction

Humans are homeothermic organisms that maintain a stable internal body temperature of ~37 °C [1]. Ambient temperature (AT) is the environmental temperature, and the thermoneutral zone is the range of ambient temperatures where the metabolic rate is relatively constant without any heat production or heat loss or regulating dry heat loss by skin blood flow. Obligatory thermogenesis maintains the body at ~37 °C under thermoneutral conditions. Obligatory thermogenesis refers to the heat produced as a byproduct of all the basal metabolic processes such as breathing, pumping of the heart, hormone homeostasis, digestion and storage of nutrients, and functioning of various ion channel proteins, etc. [2]. The thermoneutral zone (TNZ) varies widely between species, for example, 32–33 °C in mice and 26–28 °C in adult humans. When the ambient temperature falls below the lowest point of the thermoneutral zone, the balance is lost between heat loss and the heat produced by obligatory thermogenesis, resulting in a fall in body temperature. 

Body weight is determined by an intricate balance between energy intake and energy expenditure [3]. Energy expenditure is governed by four major facets, including the basal metabolic rate (obligatory thermogenesis), physical activity, nonexercised activity thermogenesis (NEAT), and adaptive thermogenesis [4]. NEAT includes daily activities such as walking around the house, gardening, etc. Adaptive thermogenesis occurs due to cold and dietary stimuli in brown and beige adipose tissue and skeletal muscles [5].

Thus, thermal energy is required to keep the body temperature stable. In general, clothing, shelter, and climate control help people maintain their body temperature within comfortable limits, and so thermoregulation is only a very small part of the daily energy expenditure. However, laboratory mice, when housed at an ambient temperature of 22–24 °C, maintain their body temperature by dietary thermogenesis, eating more food compared to thermoneutrality [6].

Mitochondrial uncoupling protein (UCP-1) is a very important component of heat production in brown adipose tissue (BAT) and inguinal white adipose tissue (iWAT) [7]. Fatty acids produced through lipolysis [8] and cold exposure-mediated adrenergic stimulation [9] result in heat generation through mitochondrial respiration via UCP-1-mediated protein leakage. Thus, it can be concluded that UCP-1 is active at ambient temperature and is inactive or less active at thermoneutral temperatures. Mice housed at ambient temperature (22 to 24 °C) experience constant cold stress, which stimulates energy intake to maintain body temperature [10]. However, thermoneutrality (~30 °C for mice) promotes adiposity despite a decrease in energy intake [11]. Surprisingly, mice that lack endogenous UCP-1 (UCP-1^−/−^) were shown to be resistant to diet-induced obesity at ambient temperature but gain weight and become obese when fed a High Fat Diet (HFD) at thermoneutrality [12]. This indicates that alternate complementary pathways mediate mechanisms of compensation due to the lack of UCP-1 at ambient temperature. However, in the TNZ, those pathways are dormant in UCP-1^−/−^ mice.

We have recently shown that the activation of the transient receptor potential vanilloid subfamily 1 (TRPV1) protein by capsaicin (CAP; a TRPV1 agonist) counters high-fat diet (HFD; 60% of calories from fat)-induced obesity without decreasing energy intake [13,14] in mice housed at ambient temperature. Our preclinical study unequivocally validates that CAP induces the browning of inguinal white adipose tissue (iWAT) and enhances the expression of genes involved in thermogenesis in iWAT and brown adipose tissue (BAT) 13,14]. The results were obtained below the thermoneutral zone in mice but in the thermoneutral zone for adult humans (21 °C) [15,16]. Therefore, the research data obtained from mice housed at 21 °C may not translate to humans, who live in thermoneutrality. 

In adipocytes, UCP-1 transcription is tightly regulated by several transcription factors. Specifically, peroxisome proliferator-activated receptor α (PPARα), PPARγ coactivator 1α (PGC-1α), and PR regulatory domain 16 containing protein (PRDM-16) are shown to regulate UCP-1 transcription [17,18,19,20]. We have delineated the mechanism by which CAP activation triggers the signal transduction pathway leading to the activation of SiRT-1, resulting in the deacetylation of two transcription factors, PPARγ and PRDM-16, and their interaction that leads to thermogenesis. Alternately, we have also shown that SiRT-1 also increased PGC-1α and PPARα levels, which increased UCP-1 expression and thermogenesis [13].

Hence, we wanted to know if CAP can induce thermogenesis and weight loss in mice housed in the thermoneutral zone (TNZ). Analyzing this is important since this will help in advancing TRPV1 activation as an effective strategy to promote weight loss in humans because humans remain in a thermal comfort zone. Furthermore, whether the effect of TRPV1 activation is dependent on the UCP-1-dependent thermogenic process remains unexplored. To address this, we evaluated the effect of CAP feeding on WT and UCP-1^−/−^ mice maintained under ambient or thermoneutral conditions and analyzed how TRPV1 activation regulates UCP-1 expression and BAT thermogenesis at thermoneutrality. Our research presents novel findings that demonstrate that TRPV1 activation by CAP at thermoneutrality counters obesity in WT mice and promotes PRDM-16-dependent UCP-1 transcription. CAP fails to inhibit weight gain in UCP-1^−/−^ mice housed at thermoneutrality. In vitro, capsaicin treatment increases UCP-1 transcription in PRDM-16 overexpressing cells. Furthermore, in mice that lack PRDM-16 in the adipose tissues, CAP fails to prevent HFD-induced obesity. Our data indicate for the first time that TRPV1 activation counters obesity at thermoneutrality permissive for UCP-1 and that the enhanced expression of other thermogenic genes and proteins is not beneficial in the absence of UCP-1. 

## 2. Results

### 2.1. TRPV1 Expression and Activity in BAT and iWAT

**Figure 1:**
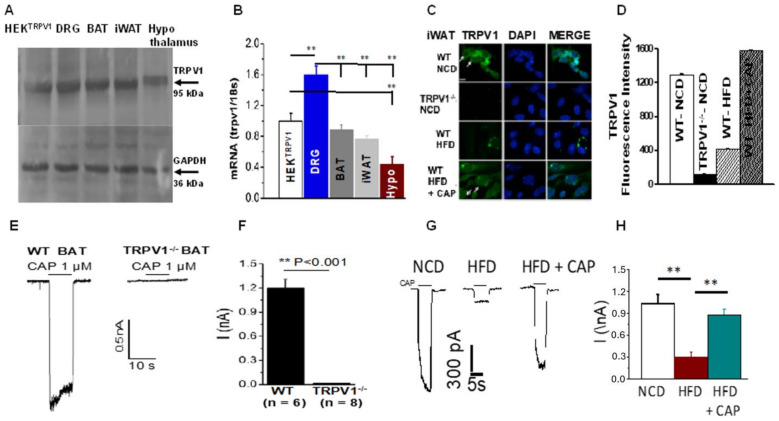
TRPV1 expression and activity in WAT and BAT. (**A**). Western blot showing TRPV1 expression in mouse tissues. HEK^TRPV1^ is the positive control. (**B**). TRPV1 mRNA expression normalized to the 18s RNA in these tissues (*n* = 5). (**C**). Representative micrograph showing the immunohistochemical detection of TRPV1 expression in the iWAT preadipocytes of WT and TRPV1^−/−^ mice fed various diets. (**D**). Quantification of the fluorescence intensity (arbitrary units). (**E**). Representative traces of CAP-stimulated TRPV1 currents in the primary brown preadipocytes of WT and TRPV1^−/−^ mice at −60 mV. (**F**). Average currents ± S.E.M. in these cells (*n* = 6 to 8). (**G**). CAP-stimulated TRPV1 currents in NCD- or HFD (±CAP)-fed primary brown preadipocytes from WT mice. (**H**). Average currents ± S.E.M. in these cells (*n* = 9 to 11). ** *p* < 0.01, significantly different.

TRPV1 expression was detected in iWAT and BAT by immunoblotting and qRT-PCR. To compare the relative expression of TRPV1 in adipose tissues, immunoblotting was performed to show that the molecular weight of TRPV1 in adipose fat corresponded to that of TRPV1 found in the dorsal root ganglion and the HEK TRPV1 stable cell line. The mRNA of TRPV1 was quantified in iWAT, BAT, DRG, and in TRPV1 overexpressing cells. Immunohistochemistry for TRPV1 in primary adipocytes was maximum under normal diet-fed conditions and was downregulated under HFD-fed conditions. CAP prevented the downregulation of TRPV1 activity under HFD-fed conditions. Furthermore, HFD suppressed the CAP-activated TRPV1 currents, and this was restored in the primary brown adipocytes of HFD + CAP-fed WT mice, as shown in Figure 1

### 2.2. Decreased Body Temperature in Diet-Induced Obese WT Mice at Ambient Temperature and in the Thermoneutral Zone

**Figure 2:**
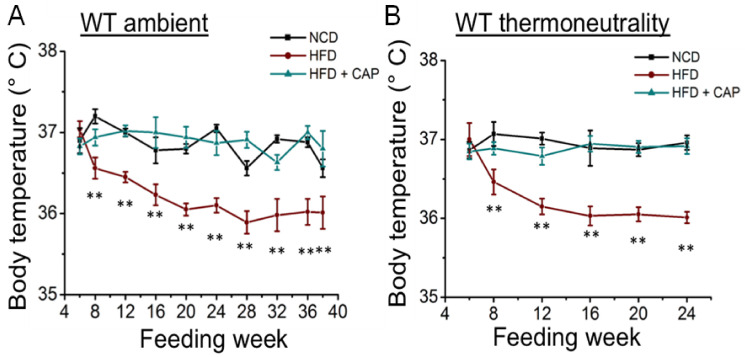
Rectal temperatures of WT mice at ambient temperature (**A**) and in the TNZ (**B**). Rectal temperatures of WT mice fed the NCD or HFD or HFD + CAP at ambient temperature and in the TNZ measured every week from 6 weeks till 38 weeks of age using a thermometer. Average mean temperatures ± S.E.Ms. of these mice (*n* = 8 for each condition). ** *p* < 0.01, significantly different.

At ambient temperature, adaptive thermogenesis is triggered by increasing food intake and heat production. However, in obese mice, a downregulation of the thermogenic machinery occurs [13,14]. So, we wanted to see the body temperature of WT mice housed at ambient temperature and in the TNZ. As shown in Figure 2, HFD-fed obese mice had a lower body temperature compared to NCD mice at both ambient temperature and in the TNZ. CAP-fed mice had body temperatures like those of NCD mice, indicating effective thermogenesis. There was no difference in body temperature of the WT mice fed the NCD and HFD + CAP at ambient temperature or in the TNZ.

### 2.3. CAP Inhibited Weight Gain in WT and UCP1^−/−^ Mice at Ambient Temperature but Inhibited HFD-Induced Weight Gain Only in WT Mice and Not in UCP^−/−^ Mice in the TNZ

**Figure 3:**
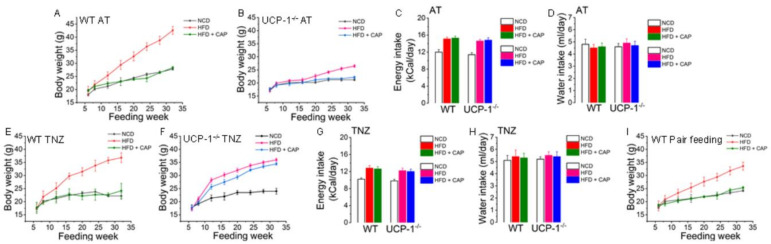
CAP counters HFD-induced weight gain in WT mice at ambient temperature and at thermoneutrality. Weight gain plotted against the feeding week for NCD- or HFD (±CAP, 0.01% in diet)-fed WT and UCP-1^−/−^ mice at ambient temperature (**A**,**B**) and at thermoneutrality (**E**,**F**). Mean energy and water intake (±S.E.M.) of these mice at ambient temperature (**C**,**D**) and at thermoneutrality (**G**,**H**). (**I**). Weight gain plotted against the feeding week for NCD- or HFD (±CAP, 0.01% in diet)-fed WT mice that received the same number of calories that WT mice received at ambient temperature.

Our earlier data show that dietary CAP significantly antagonizes diet-induced obesity at ambient temperature by activating UCP-1 [10]. As shown in Figure 2, there was no difference in body temperature between CAP-fed mice at ambient temperature and in the TNZ. So, we wanted to study the body weight gain of WT mice and UCP-1^−/−^ mice at ambient temperature and in the TNZ. The body weights were the same between all the cohorts at the beginning of the feeding experiments at 6 weeks of age (Figure 3A,B,E,F). The weight gain became statistically different when the WT mice gained weight at a faster rate compared to UCP-1^−/−^ mice. WT mice fed the HFD weighed twice as much as UCP-1^−/−^ mice fed the HFD at ambient temperature (Figure 3A,B). CAP-fed mice weighed the same as their NCD-fed counterparts. There was no difference in food or water intake between the different cohorts. The HFD increased weight gain in UCP-1^−/−^ mice housed in the TNZ, and CAP failed to inhibit the weight gain of UCP-1^−/−^ mice in the TNZ (Figure 3F). There was no difference in food or water intake between WT and UCP-1^−/−^ mice fed the respective diets at ambient temperature (Figure 3C) or in the TNZ (Figure 3G). The food intake of all cohorts was slightly higher at ambient temperature compared to the TNZ (Figure 3C,G) and water intake was slightly higher in the TNZ (Figure 3D,H) compared to ambient temperature. Weight gain was less in mice with pair feeding compared to the mice that were fed ad libitum (Figure 3A,I).

### 2.4. Metabolic Activity

**Figure 4:**
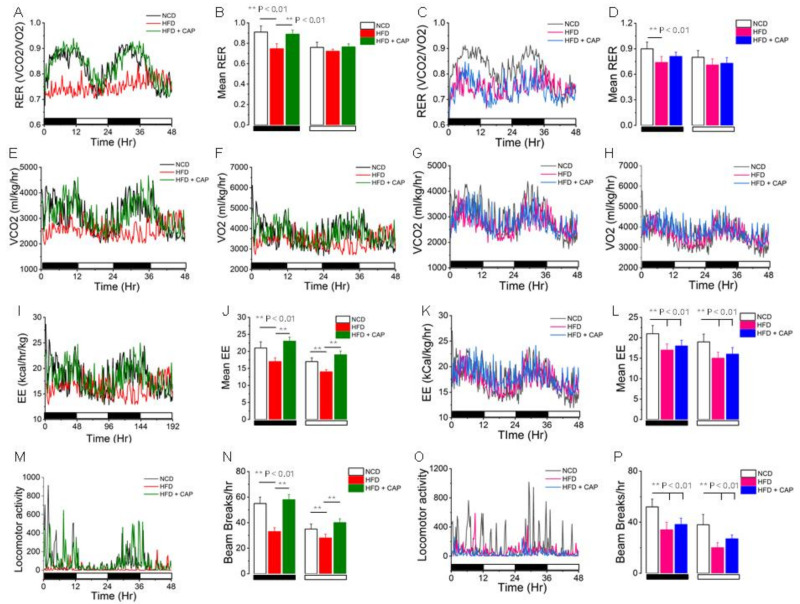
CAP feeding increases the respiratory quotient (respiratory exchange ratio, RER= VCO2/VO2) and energy expenditure (EE) in WT mice maintained at thermoneutrality. RER (**A**,**C**), VCO2 (**E**,**G**), VO2 (**F**,**H**), EE (**I**,**K**), and locomotor activity (**M**,**O**) of NCD- or HFD (± CAP, 0.01% in diet)-fed WT and UCP-1^−/−^ mice at thermoneutrality. Means ± S.E.Ms. for the RER (**B**,**D**), EE (**J**,**L**), and locomotor activities (**N**,**P**) of these mice. ** represents statistical significance at *p* < 0.01 for *n* = 8 mice for each condition.

The respiratory coefficient was used to determine the basal metabolic rate. We have shown earlier that CAP increases the respiratory rate and energy expenditure of WT mice housed at ambient temperature [14,21]. So, we wanted to determine if CAP increases the respiratory rate in WT mice housed in the TNZ. The metabolic activity was determined using the Comprehensive Laboratory Animal Monitoring System at ambient temperature. The HFD inhibited the RER, energy expenditure, and locomotion. CAP revived the RER, mean energy expenditure, and locomotion in WT mice similar to the mice under the NCD-fed condition (Figure 4A,B,E,F,I,J,M,N). CAP failed to revive the respiratory coefficient, energy expenditure, and locomotion in UCP1^−/−^ mice housed in the TNZ (Figure 4C,D,G,H,K,L,O,P).

### 2.5. White Adipogenic and Thermogenic Gene Expression

**Figure 5:**
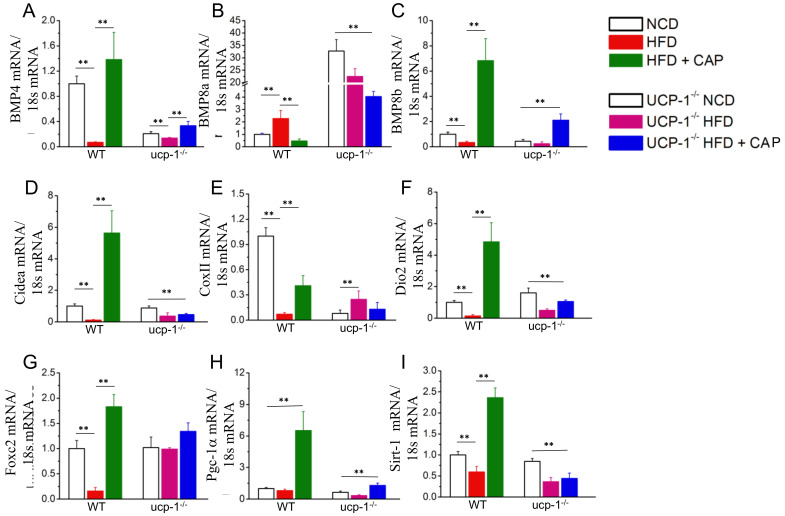
Effect of CAP feeding on the mRNA levels of adipogenic and thermogenic genes in the BAT of NCD- or HFD (±CAP)-fed WT and UCP-1^−/−^ mice at thermoneutrality. Mean mRNA levels ± S.E.Ms. for Bmp4 (**A**), Bmp8a (**B**), Bmp8b (**C**), Cidea (**D**), CoxII (**E**), Dio2 (**F**), Foxc2 (**G**), Pgc-1α (**H**), and Sirt-1 (**I**) in the BAT of these mice. For quantitative RT-PCR experiments, 18s ribosomal RNA was used as control. ** represents statistical significance at *p* < 0.01 for *n* = 4 experiments.

Since CAP inhibited the weight gain in WT mice in the TNZ similar to ambient temperature, white adipogenic and thermogenic gene expression were characterized by quantitative RT-PCR [14]. A gene expression analysis was also performed in BAT from UCP-1^−/−^ mice housed in the TNZ. Mitochondrial thermogenic genes such as BMP4, Cidea, CoxII, Foxc2 and Dio2 (Figure 5A,D,E,F,G), the mitochondrial quality control protein PGC-1alpha (Figure 5H), and energy sensor and transcriptional regulator of PGC-1alpha SiRT-1 (Figure 5I) were upregulated by CAP in WT mice, and a significant upregulation compared to WT mice was not found in UCP-1^−/−^ mice. However, the expression of BMP8a (Figure 5B), another regulator of adipocyte differentiation, was enhanced in BAT from UCP-1^−/−^ mice compared to BAT from WT mice.

### 2.6. UCP-1 and PRDM-16 Gene Expression

**Figure 6:**
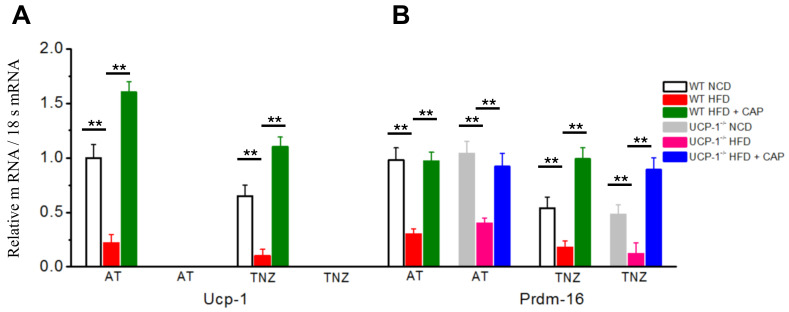
Effect of CAP feeding on the Ucp-1 (**A**) and Prdm-16 (**B**) mRNAs normalized to 18s RNA in NCD- or HFD (± CAP)-fed WT and UCP-1^−/−^ mice. Mean Ucp-1 and Prdm-16 mRNA levels normalized to 18s RNA ± S.E.Ms in the BAT of these mice. ** represents statistical significance at *p* < 0.01 for *n* = 4 experiments.

Our earlier studies have shown that CAP inhibits weight gain in diet-induced obese mice by upregulating UCP-1. To examine the involvement of UCP-1 and its transcriptional regulator PRDM16, quantitative RT-PCR was performed in the BAT of WT and UCP-1^−/−^ mice housed in the TNZ. Figure 6A shows UCP1 levels less in the TNZ compared to ambient temperature. CAP increased UCP-1 levels at both ambient temperature and in the TNZ. The UCP-1 mRNA was undetected in UCP-1^−/−^ BAT. Figure 6B shows similar PRDM16 mRNA levels in WT and UCP-1^−/−^ BAT at ambient temperature, as well as in the TNZ. PRDM16 was downregulated under HFD-fed conditions across the conditions and CAP increased the mRNA levels in WT and UCP-1^−/−^ BAT at both AT and in the TNZ.

### 2.7. CAP Fails to Counter HFD-Induced Weight Gain in Adipose Tissue-Specific PRDM16^−/−^ Mice

**Figure 7:**
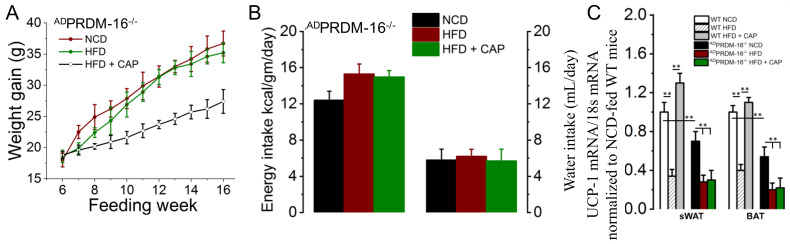
CAP does not counter obesity in ^AD^PRDM-16^−/−^ mice. (**A**) Mean body weight gain in NCD- or HFD (± CAP, 0.01% in diet)-fed ^AT^PRDM-16^−/−^ mice (*n* = 4). (**B**) Average daily energy and water intake in these mice (*n* = 6). (**C**) UCP-1 mRNA levels in the sWAT and BAT of these mice (*n* = 6 experiments). ** *p* < 0.01.

To confirm that CAP exerts its thermogenic activity through the PRDM16-UCP-1 pathway, adipose tissue-specific PARDm16^−/−^ mice were generated through the Cre-lox system by crossing PRDM16 lox mice with FABP4-cre mice. The adipose tissue-specific PRDM16 knockout mice gained weight with the HFD, which was not inhibited by feeding CAP at ambient temperature, as shown in Figure 7A. There was no difference in food or water intake between the HFD and HFD + CAP cohorts. Figure 7C shows the UCP-1 mRNA levels in BAT of adipose tissue-specific PRDM16^−/−^ mice. UCP-1 mRNA levels were low in ^AD^PRDM16^−/−^ mice fed different diets in comparison to the respective WT controls.

### 2.8. PRDM-16 Overexpression Upregulates UCP-1 Transcription in HEK TRPV1 Cells

**Figure 8:**
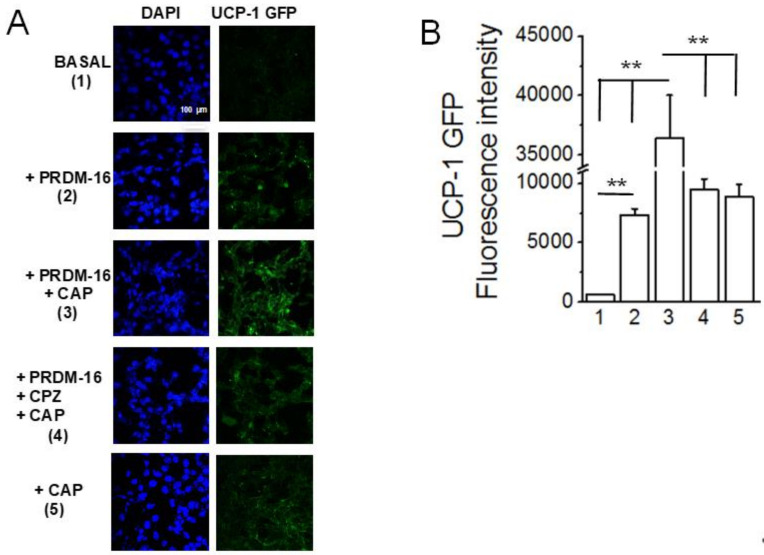
PRDM-16 overexpression increases UCP-1 transcription and CAP treatment enhances it further. (**A**) Micrographs showing UCP-1-GFP expression in HEK TRPV1 cells from the control (basal; 1), PRDM-16 (2), PRDM-16 + CAP (1 μM; 3), PRDM-16, CPZ (10 μM; TRPV1 inhibitor) + CAP (1 μM), or CAP (1 μM) treatment groups. The magnification is 10x, and the scale bar is 100 μm. (**B**) Mean intensity of UCP-1-GFP normalized to the control (basal) group ± S.E.M. for *n* = 3 independent experiments. ** *p* < 0.01.

As the knockout of PRDM16 resulted in a decrease in UCP-1, the effect of overexpression of PRDM16 on UCP-1 was analyzed in stable TRPV1-expressing HEK293 cells. As shown in Figure 8, CAP stimulated the GFP reporter-driven UCP-1 promoter. PRDM16 co-transfection increased the expression of GFP, which was further stimulated by CAP and inhibited by the TRPV1 antagonist capsazepine.

## 3. Discussion

Obesity is excess adiposity and plays a central role in cellular metabolic dysregulation that accounts for insulin resistance, type 2 diabetes, dyslipidemia, hypertension, atherosclerosis, fatty liver, etc. [22]. Obesity is a serious public health problem affecting more than two of five adults in the United States [23]. Recent research has undoubtedly shown the presence of BAT in humans, and its activation inhibits insulin resistance and obesity[24]. BAT is active in mice housed at ambient temperature. Humans generally live in the TNZ without expending extra energy to maintain normal body temperature. This work systematically analyzed the role of TRPV1 activation in countering obesity in WT mice housed in the TNZ and delineated the mechanism of UCP1 activation in energy expenditure in the TNZ using WT and UCP-1^−/−^ mice.

This research demonstrates that TRPV1 is expressed in both iWAT and BAT and its expression is inhibited by HFD (Figure 1). Furthermore, CAP counters this, which is consistent with previous findings [13,14]. In addition, we have previously shown that TRPV1 activation in BAT and iWAT triggers signal transduction pathways involving SiRT-1, which causes deacetylation of PPAR gamma and PRDM-16 and stimulates their interaction in iWAT to cause the browning of iWAT [13,14]. However, these experiments were performed by housing mice at ambient temperature. These mice experienced mild cold stress at ambient temperature. However, chronic HFD feeding resulted in a defective thermogenic capacity, leading to obesity. In the HFD + CAP-fed group, TRPV1 activation by CAP increased UCP-1 expression and activity and converted fat to heat. However, this research did not show how TRPV1 activation enhanced UCP-1 expression and activation.

We hypothesized that CAP stimulates PRDM-16 to enhance UCP-1 expression transcriptionally. Consistent with this, CAP failed to increase UCP-1 expression in the BAT of adipose-specific PRDM16 KO mice, while it enhanced UCP-1 mRNA expression in the BAT of WT mice (Figure 7). Furthermore, overexpression of PRDM-16 in stable TRPV1-expressing HEK293 cells induced an increase in UCP-1 transcription, which was further enhanced by CAP treatment (Figure 8) These data unequivocally demonstrate that TRPV1 activation by CAP enhances UCP-1 expression via PRDM-16. 

Uncoupling proteins are anchored in the inner membrane of mitochondria and take part in the maintenance of the energy balance by diverting energy from ATP synthesis to thermogenesis [25]. They are highly expressed in brown and beige adipocytes, which are rich in mitochondria. UCP-1, also called thermogenin, is responsible for BAT thermogenesis and systemic energy homeostasis through excessive energy intake [26] and the generation of body heat through uncoupling oxidative phosphorylation during adaptive thermogenesis [27]. Thus, this indicates the significance of UCP-1 in preventing excessive adiposity in mice. Accordingly, UCP-1 KO mice develop hypothermia when exposed to cold temperatures [28]. However, in contrast, UCP-1 KO mice did not gain weight on the HFD when housed at ambient temperature. They also showed increased locomotion and oxygen consumption and a reduced respiratory coefficient, indicating alternate pathways for the metabolism of lipids and maintaining body temperature [29]. However, in the thermoneutral zone, UCP-1 activation is not required to maintain body temperature, and hence UCP-1 and alternate pathways of lipid metabolism remain dormant. Thus, UCP-1 has to be activated to produce heat [30]. Consistently, UCP-1 KO mice gained body weight after feeding on the HFD in the TNZ, as the alternate pathways for thermogenesis were not triggered (Figure 3). Thus, UCP-1 activation is required to maintain energy homeostasis under high energy intake conditions at thermoneutrality.

We have earlier shown that the HFD diet inhibited the expression and activity of thermogenic genes, including UCP-1, in both BAT and iWAT. Dietary CAP inhibited diet-induced obesity by increasing thermogenesis. Thus, HFD diet-induced obese mice showed a reduced body temperature at both ambient temperature, as well as in the TNZ, where normal chow diet-fed mice and mice with dietary CAP treatment maintained a body temperature of 37 °C (Figure 2). CAP inhibited weight gain in UCP1^−/−^ mice at ambient temperature. This was not significant, as the HFD did not cause weight gain in UCP1^−/−^ mice at ambient temperature (Figure 3A,B). However, CAP inhibited weight gain in WT mice in the TNZ but not in UCP1^−/−^ mice (Figure 3E,F). This shows that CAP increases diet-induced thermogenesis by activating mitochondrial UCP-1 in BAT and iWAT. Similarly, UCP-1 is required for CAP-mediated increases in oxygen consumption, RER, and energy expenditure (Figure 4).

Thermogenic genes such as BMP4, BMP8b, Cidea, COX II, Dio2, FoxC2, PGC1-α and SiRT-1 are either directly or indirectly involved in the transcription of UCP-1 and therefore thermogenesis [31,32,33,34,35,36,37,38]. There was no or a modest upregulation of these thermogenic genes by CAP in UCP-1^−/−^ mice in the TNZ (Figure 5). This suggests that the lack of UCP-1 in the BAT of UCP-1 KO mice suppresses these thermogenic genes through unknown mechanisms. Interestingly, CAP increases PRDM-16 expression in the UCP-1 KO mice (Figure 6). However, this does not enhance metabolic activity or heat production (Figure 4). Thus, our results confirm that the mechanisms of action of CAP to counter HFD-induced obesity are permissive of UCP-1 in adipose tissues. 

PRDM16 is a critical regulator of UCP-1 transcription [20]. PRDM16 suppresses the expression of white fat-specific genes in BAT and maintains the brown fat-specific profile in iWAT. Therefore, mice lacking PRDM16 specifically in adipose tissues will have substantially less heat production [39,40]. Dietary CAP upregulated the PRDM16 mRNA to the same extent in WT and UCP-1^−/−^ mice at ambient temperature and in the TNZ (Figure 6B). The HFD inhibited the PRDM 16 in both WT and UCP1^−/−^ mice at ambient temperature and in the TNZ. However, the UCP-1 mRNA was detected only in WT mice, and it was higher at AT than in the TNZ (Figure 6A). These data collectively suggest a regulatory role for PRDM 16 in UCP-1 transcription and therefore energy homeostasis. The regulatory role of PRDM 16-mediated UCP-1 transcription was further confirmed in Figure 7. In adipose tissue-specific PRDM16 knockout mice, CAP failed to inhibit HFD-induced weight gain and did not increase UCP-1 mRNA levels. Similarly, CAP stimulation in PRDM16-overexpressing cells increased UCP-1 gene expression (Figure 8).

**Figure 9:**
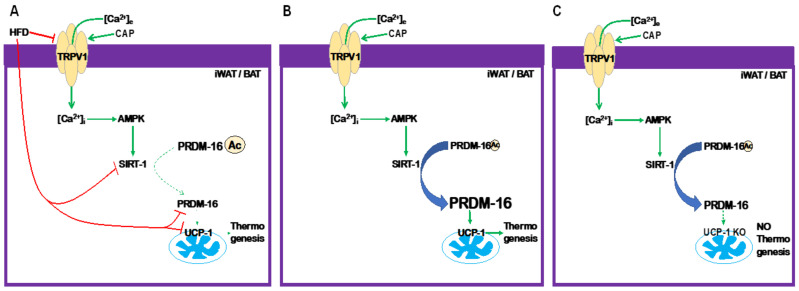
Model describing the effect of TRPV1 activation by CAP on PRDM-16-dependent UCP-1 expression and therefore thermogenesis. (**A**). The HFD inhibits TRPV1 [10], SiRT-1, and PRDM-16 [9]. Inhibition of SiRT-1 suppresses the deacetylation of PRDM-16 [9] and PRDM-16-dependent UCP-1 transcription. (**B**). CAP counters the effect of the HFD. (**C**). In UCP-1 KO mice, CAP activates the TRPV1-SIRT-1-PRDM-16-dependent signaling axis, but it fails to stimulate thermogenesis.

Thus, our data unambiguously demonstrate that CAP counters diet-induced obesity in WT mice robustly at ambient temperature and in the TNZ through the PRDM16-mediated enhancement of UCP-1 transcription, as shown in Figure 9B. The HFD inhibits TRPV1, prevents Ca2+ influx and activation of kinases, and activates SIRT-1. This results in the acetylation of PRDM-16 and its downregulation, as shown in Figure 9A. HFD-induced obesity is associated with a diminished ability to expend energy as heat due to the downregulation of UCP-1, as PRDM16 is downregulated. As shown in Figure 9C, the CAP-mediated signal transduction mechanism maintains PRDM-16 in a deacetylated state but energy is not expended, as UCP-1 is not present in UCP-1^−/−^ mice. Our data provide strong evidence that the TRPV1/PRDM-16/UCP-1 axis inhibits weight gain at ambient temperature, as well as in the TNZ (model shown in Figure 9). These data have a significant translational impact since humans protect themselves from external variations in temperatures and remain in a thermoneutral zone, and targeting and activating TRPV1 at thermoneutrality in humans would be a viable strategy to counter diet-induced obesity. Thus, this research reveals novel and promising findings to support the activation of TRPV1 in humans.

## 4. Materials and Methods

### 4.1. Mouse Model of High-Fat Diet-Induced Obesity

Adult male and female UCP-1^−/−^ (129S-Ucp1*^tm^^1Kz^*/J) mice and their genetically unaltered control (wild type, WT) mice were purchased from the Jackson Laboratory, Maine, USA. Mice were housed in the research animal facility at the School of Pharmacy, University of Wyoming, and used for experiments as per approved IACUC protocols. UCP-1^−/−^ mice were genotyped three weeks after birth using primers specified by Jackson Laboratories. 

For experiments, groups of WT and UCP-1^−/−^ mice were fed a normal chow diet (NCD) until six weeks of age and then fed ad libitum either the NCD (Labdiet Rodent 5001; gross energy kcal/g is 4.07) or were switched to a high-fat diet (HFD; 60% calories from fat; D 12492; Research Diet Inc., New Brunswick, NJ, USA; 5.21 kcal/g and fed until 38 weeks of age. Mice were randomly assigned into feeding groups and were housed in groups of four in separate cages. Mice were maintained either at ambient temperature (22–24 °C) or at thermoneutrality (29 to 31 °C) on a twelve-hour dark–light cycle and their weekly weight gain and food and water intake were recorded in a blinded fashion. For energy/water intake, the average quantity consumed per mouse per day was calculated, and the mean values of data were pooled from week 6 through week 38 of feeding. At the end of 32 weeks of feeding (mice were 38 weeks of age), a metabolic study was performed with a group of mice. Two days after the completion of the metabolic study, epididymal and brown adipose fat pad tissues (EF and BF, respectively) were isolated and used for quantitative RT–PCR experiments.

### 4.2. Adipose Tissue-Specific PRDM-16 Knockout Mice

Adipose tissue-specific PRDM16 knockout mice were generated by crossing PRDM16^lox/lox^ mice with Fabp4-cre mice. Genotyping was carried out using primers specified by Jackson Laboratories (Bar Harbor, ME, USA) for generic Cre.

### 4.3. Measurement of Rectal Temperature in Mice

The rectal temperature was determined by inserting a petroleum jelly-lubricated thermometer into the rectum of the mice. The temperature was recorded when it was stable, and the probe was removed.

### 4.4. Metabolic Activity Measurement

Metabolic activity and the respiratory quotient were determined for NCD- or HFD-fed WT and TRPV1^−/−^ mice at the end of 32 weeks of feeding using the Comprehensive Laboratory Animal Monitoring System [CLAMS™], Columbus Instruments, Columbus, OH, USA [41,42]. Mice were individually placed in the CLAMS metabolic cages with ad libitum access to food and water. After acclimatization for 48 hr., metabolic parameters, including the volume of carbon dioxide produced (VCO2), the volume of oxygen consumed (VO2), the respiratory quotient (RQ), and respiratory exchange ratio (RER = VCO2/VO2), and ambulatory/locomotor activity were determined as described previously. 

### 4.5. Fat Tissue Isolation [43]

Following euthanization, mice were placed on a surgical pad with the dorsal surface facing up and cut open along the back to the neck. The intrascapular BF found right under the skin between the shoulders, seen as two lobes, was dissected.

For inguinal subcutaneous fat pad isolation, a cut was made through the skin using a scalpel just below the rib cage across the dorsal surface joining the two lateral incisions. After peeling the skin, the fat underlying the muscle and fascia was dissected using a pair of scissors.

### 4.6. Isolation and Primary Culture of Brown Adipose Tissue Preadipocytes

The primary preadipocyte culture from BAT was performed in the same way as from SCF, as described previously [14].

### 4.7. Immunohistochemistry

Immunostaining for TRPV1 and DAPI was performed in primary adipocytes obtained from the iWAT of NCD-, HFD-, or HFD + CAP-fed WT mice, as described previously [14].

### 4.8. Whole-Cell Patch Clamp of Primary Brown Adipocytes

TRPV1 activity was measured at -60 mV in the primary brown preadipocytes by measuring CAP (1 μM)-stimulated currents using patch clamping, as described previously [44].

### 4.9. Quantitative RT-PCR Measurements (qRT-PCR)

The isolation of total RNA was performed using Tri-reagent (Sigma, St. Louis, MO, USA) as per the manufacturer’s protocol. cDNA was synthesized using a QuantiTect reverse transcription kit (Qiagen, Valencia, CA, USA) using a Q5plex PCR system (Qiagen, Valencia, CA, USA). Real-time PCR was performed using a QuantiTect SYBR Green PCR kit on the Q5plex system. 18s RNA was used as the internal control. Amplifications were performed in a 25 µL reaction volume. All the primers used for quantitative RT-PCR experiments are given in Table 1. 

### 4.10. Immunoblotting

Isolated fat pads were washed with chilled PBS, lysed in lysis buffer (50 mM, Tris pH 7.5, 250 mM sodium chloride, 0.5% NP40, 0.5% sodium deoxycholate, 2 mM EDTA, 0.5 mM dithiothreitol, 1 mM sodium orthovanadate, and protease inhibitor cocktail), and centrifuged at 14000 rpm for 20 min. to remove tissue debris. The concentration of protein was determined by the Bradford method and equal amounts of protein were separated by SDS-PAGE, transferred to a nitrocellulose membrane, and immunoblotted with a TRPV1 antibody at a dilution of 1:100, (SC-28759, Santacruz Biotechnologies, Dallas, TX, USA) and a GAPDH antibody at a dilution of 1:1000, (SC-365062, Santacruz Biotechnologies, Dallas, TX, USA).

### 4.11. PRDM16 and GFP-UCP1 Overexpression and Stimulation in the HEK TRPV1 Cell Line

The HEK TRPV1 cells were grown to 70% confluence in poly-lysine-coated plates and were transfected with UCP-1-GFP alone (#104585, Addgene, Watertown, MA 02472, USA) or UCP-1-GFP and PRDM16 (Origene, #MC229536, Rockville, MD 20850, USA) using Lipofectamine transfection reagent. UCP-1-GFP-transfected cells were treated with vehicle or CAP (1 μM) overnight. UCP-1-GFP- and PRDM16-transfected cells were treated with vehicle or CAP (1 μM) or capsazepine (10 μM) for 1 h followed by CAP and measured for GFP fluorescence using a confocal microscope.

### 4.12. Drugs and Chemicals

The normal chow diet was obtained from Lab Diets, Inc., USA. Other special diets were obtained from Research Diets Inc., New Brunswick, NJ, USA. All chemicals were obtained from Sigma, USA. Quantitative RT-PCR kits were obtained from Qiagen, USA. 

### 4.13. Statistical Analyses

Data for all figures are expressed as means ± S.E.Ms. Student’s *t*-test and ANOVA evaluated the statistical significance of population means. Sample sizes were set to determine whether the mean value of an outcome variable in one group differed significantly from that in another group. A *p* value < 0.05 was considered statistically significant. Comparisons between groups were analyzed using one-way ANOVA, and post hoc analyses were performed using Tukey’s test. Figures from analyzed data were plotted using Microcal Origin 2020 software (OriginLab, Northampton, MA, USA), and figures were generated using the same program and then converted into image files using the software Adobe Photoshop CS5 Extended (version 12.1; www.adobe.com).

## Figures and Tables

**Table 1:** Quantitative RT-PCR primers used for the research study.

Gene (Accession Number)	Forward Primer	Reverse Primer
*18s (X00686)*	*accgcagctaggaataatgga*	*gcctcagttccgaaaacca*
*Gapdh (NM_001411844*)	*cgtgccgcctggagaaacc*	*tggaagagtgggagttgctgttg*
*mtrpv1* (NM_001001445)	*caacaagaaggggcttacacc*	*tctggagaatgtaggccaagac*
*sirt-1 (NM_019812)*	*tcgtggagacatttttaatcagg*	*gcttcatgatggcaagtgg*
*prdm-16* (XM_03616442)	*cagcacggtgaagccattc*	*gcgtgcatccgcttgtg*
*ucp-1 (NM_009463)*	*cgactcagtccaagagtacttctcttc*	*gccggctgagatcttgtttc*
*pgc-1α (NR_175325)*	*agagaggcagaagcagaaagcaat*	*attctgtccgcgttgtgtcagg*
*Cidea (NM_007702)*	*atcacaactggcctggttacg*	*tactacccggtgtccatttct*
*cox2 (AF378830)*	*ataaccgagtcgttctgccaat*	*tttcagagcattggccatagaa*
*foxc2 (NM_013519)*	*gcaacccaacagcaaactttc*	*gacggcgtagctcgatagg*
*dio2 (NM_010050)*	*gttgcttctgagccgctc*	*gctctgcactggcaaagtc*
*bmp4* *(NM_007554)*	*ctccagtctggggaggag*	*gatgaggtgcccaggcac*
*bmp8a (NM_007558)*	*aaccatgccatcttgcagtct*	*cagaggtggcactcagtttgg*
*bmp8b (NM_007559)*	*tccaccaaccacgccactat*	*cagtaggcacacagcacacct*

## Data Availability

Data is contained within the article.
